# Phytonutrient and Nutraceutical Action against COVID-19: Current Review of Characteristics and Benefits

**DOI:** 10.3390/nu13020464

**Published:** 2021-01-30

**Authors:** Nitida Pastor, Maria Carmen Collado, Paolo Manzoni

**Affiliations:** 1 Department of Medical Affairs, Clinical Research, Mead Johnson Nutrition, Evansville, IN 47721, USA; 2 Department of Biotechnology, Institute of Agrochemistry and Food Technology-National Research Council (IATA-CSIC), Agustin Escardino 7, 46980 Paterna, Valencia, Spain; mcolam@iata.csic.es; 3 Division of Pediatrics and Neonatology, Department of Maternal, Neonatal, and Infant Medicine, Nuovo Ospedale Degli Infermi, 13875 Biella, Italy; paolomanzoni@hotmail.com; 4 Neonatology and NICU, Sant’Anna Hospital, AOU Città della Salute e della Scienza, 10136 Torino, Italy

**Keywords:** phytonutrients, nutraceutical, flavonoids, immune system, COVID-19, SARS-CoV-2

## Abstract

The trend toward using phytonutrients and/or nutraceuticals (P/Ns) with the aim of impacting immune health has increased in recent years. The main reason is that properties of P/Ns are associated with possible immunomodulating effects in the prevention and complementary treatment of viral diseases, including COVID-19 and other respiratory infections. In the present review, we assess the scientific plausibility of specific P/Ns for this purpose of preventative and therapeutic interventions against COVID-19, with an emphasis on safety, validity, and evidence of efficacy against other viruses. Five potential candidates have been identified after reviewing available studies (in silico, in vitro, and in vivo) in which certain flavonoids have demonstrated a potential for use as adjuvant therapeutic agents against viral infections, including COVID-19. As these are often better tolerated than pharmacological treatments, their use could be more widely considered if additional detailed studies can validate the existing evidence.

## 1. Introduction

The terms phytonutrients and nutraceuticals are highly interconnected and have both been used widely in the scientific, regulatory, and popular literature. The term “nutraceutical” was established in 1989, combining the words “nutrition/nutrients” (a nourishing food component) and “pharmaceutical” (medicine or a substance used as a medication) and implying use for prevention and/or treatment of disease. The term “phytonutrient” comes from the Greek word phyton (plant) and refers to substances derived from, or identical to, those occurring in plants. Definitions of these two entities are still in progress, and in practice, both terms are often used interchangeably, although this is in principle not entirely correct. Based on the latest evidence, a working definition for phytonutrient could be a compound present in and/or derived from plants that confer a health benefit, including metabolites after consumption. A nutraceutical can be defined as a compound or a mixture of compounds present in food or food supplements intended to exert a therapeutic effect; the source could be plant or animal [1]. 

In both categories, the most highly represented compounds are polyphenols, which can be divided into phenolic acids and flavonoids. The largest groups of naturally occurring flavonoids include flavones, flavonols, flavanones, flavanols, isoflavonoids, and anthocyanidins. Flavonoids are characterized by broad biological activities as demonstrated in numerous mammalian systems in vitro and in vivo. These compounds act as free-radical scavengers and antioxidants, exhibiting antimutagenic, anti-inflammatory, cardiovascular, tumorigenic process, immune, and antiviral effects [2]. Flavonoids are present in vegetables and fruits such as onions, grapes, apples, and cherries [3]. 

In ethnobotany, traditional Chinese medicine (TCM), and Ayurvedic medicine, medicinal herbs and extracts have been used for generations as they appear to often have positive impacts on health. These treatments remain fashionable today. However, it is less clear whether these practices have a beneficial impact on viral infections, either in prevention or adjuvant treatment, and what possible mechanisms may be involved. 

Previous clinical data with other viruses and newer in silico modeling can be used to guide the identification of potential candidates for future trials of preventative or antiviral nutraceutical/phytonutrient treatment. For example, previously reported benefits of TCM for SARS-CoV (severe acute respiratory syndrome) in 2004 showed that the duration of main symptoms in the group of patients treated by Western medicines alone stayed longer in comparison with the group of patients treated with integrated Chinese and Western medicines. In a recent retrospective cohort study regarding the treatment of COVID-19, this integration had benefits including symptomatic relief, shortened fever duration, improved radiological changes, and shortened hospital stay [4]. Furthermore, the clinical evidence generated from SARS and H1N1 management can support hypothesized mechanisms and identify potential flavonoids that could be considered candidates for concomitant therapy for COVID-19.

A recent study conducted in Wuhan, China, identified a positive relationship between the TCM concept of “invigorating spleen and removing dampness” and improvement in novel coronavirus pneumonia (NCP), suggesting the importance of regulating intestinal function and microenvironmental balance [5]. In that study, TCM compounds such as quercetin, luteolin, or kaempferol were included in the treatment. 

The potential uses of medicinal herbs and their extracts are linked to understanding their different mechanisms of action, thereby suggesting a potential use in either prevention or treatment or as an adjuvant in the recovery phase [3,6,7]. One of the most intriguing pathways consists of blocking the angiotensin-converting enzyme (ACE) since it is critical for SARS-CoV-2 adhesion to cells. It has therefore been speculated that two proteins, 3C-like protease (3CLpro) and angiotensin-converting enzyme 2 (ACE2), could be used as targets to screen drugs for their ability to inhibit replication and proliferation of SARS-CoV-2 [8]. More specifically, the use of the enzyme asparaginase to target the terminal amino acid asparagine of the ACE2 peptide, thus preventing adhesion of the virus, is currently under investigation (Manzoni, unpublished data). 

The main SARS-CoV-2 protease (Mpro)/chymotrypsin-like (3CLpro) represents a target to inhibit viral replication. Functionally, Mpro is highly conserved among coronaviruses. Sequence alignment revealed that the Mpro of SARS-CoV-2 shares 96% similarity with that of SARS. Protein modeling and virtual screening have been used to identify 30 Mpro inhibitors [9], particularly those with potential anti-inflammatory properties or the ability to limit cytokine storm. Studies based on analyzing binding energies from the docking with a native ligand with specific plants and extracts have shown properties to defend against viral infection. Flavonoids represent a category of ingredients most commonly proposed to have antiviral activities based on in silico models [10,11]. Finally, the potential activity of Lianhuaqingwen, a Chinese nutraceutical mixture composed of 13 herbs, has been tested against the SARS-CoV-2 virus in vitro. In addition to inhibition of viral replication, reduction of proinflammatory cytokines, including TNF-α, IL-6, CCL-2/MCP-1, and CXCL-10/IP-10, was also demonstrated [12].

Overall, the literature is relatively scant on the topic of possible P/N use in viral infections, and even more specifically in COVID-19 infection. Most publications are based on extrapolations for potential use based on previous experience with another virus, such as the earlier SARS (severe acute respiratory syndrome) coronavirus. Often, the best available evidence is based on in silico and in vitro studies. For many herbal extracts, little or no data exist on efficacy, side effects, dosage, or drug interactions. Among natural plant compounds, the most suitable candidates to act as Mpro inhibitors are curcumin, kaempferol, quercetin, and apigenin (as represented in elderberry, *Echinacea*, *Matricaria*, parsley, and celery) [10,12,13,14]. 

Based on these considerations, the objective of this paper is to review P/Ns in terms of putative antiviral use and proposed mechanisms of action. We aim to assess the scientific plausibility of specific compounds in the prevention and complementary treatment of viral diseases, particularly COVID-19. Safety, validity, and efficacy must be demonstrated prior to a specific P/N recommendation for such purposes.

## 2. Review

A literature review was carried out using Embase, Scopus, PubMed, Google Scholar, and ASD-related electronic databases for each of the search terms listed below, as well as the term “nutraceuticals”:

▪ Nutrient/ingredient-related terms: ascorbic acid, vitamin D, zinc, quercetin, kaempferol, curcumin demethoxycurcumin, turmeric, turmeric extract, turmeric oil, capper, garlic, apigenin, naringenin, echinacea, echinacea extract, echinacea purpurea, echinacea purpurea extract, echinacea angustifolia extract, and pelargonium sidoides extract.
▪ Viral-related terms: immunity, antiviral activity, virus entry, virus pathogenesis.
▪ COVID-related terms: coronaviridae, coronaviridae infection, coronavirus infection, COVID-19.

In addition, specific searches on identified phytonutrients were carried out to complement the review. References were limited to the English language and to a publication date of 2010 to the present day with exceptional selections. Studies reporting in silico, in vitro, in vivo, animal, or human data were included. 

## 3. Results

With these search criteria, we identified five potential candidates that are described below and summarized in Table 1 and Table 2.

### 3.1. Curcumin (Curcuma longa)

Background: The dried rhizome of *Curcuma longa* is the source of turmeric, the widely consumed spice used in foods and in Ayurveda medicine. Curcumin (E,E-1,7-bis-(4-hydroxy-3-methoxyphenyl)-1,6-heptadiene-3,5-dione), is the yellow pigment extracted from the rhizomes. It exhibits several pharmacologic properties, such as antioxidant, anti-inflammatory, and antifibrotic properties. It is important to note that not all the analogs are the same, and their stability and efficacy are dose-dependent and related to a specific type [15,16,17].

Evidence: In vitro and in vivo animal studies have shown that curcumin is active against different viruses, bacteria, fungi, and emerging and multidrug-resistant strains [18,19]. The turmeric-derived polyphenol curcumin has been tested for therapeutic potential against SARS-CoV-2 (based on potential anti-HIV activity due to HIV-1 or HIV-2 protease inhibitor and HIV-1 integrase inhibitor activities, possible inhibition of SARS-CoV-2 has been suggested). It has also been shown that Zika and chikungunya virus incubated with curcumin lose their infectivity [16]. The potential mechanism of action relies on the reduction of the protein level of the Ang II type 1 (AT1) receptor and upregulation of the Ang II type 2 (AT2) receptor, evidenced by an increased ratio of AT2 receptor to AT1 receptor in the curcumin group (1.2 ± 0.02%) vs. the Ang II group (0.7 ± 0.03%, *p* = 0.05). Additionally, curcumin has been shown to decrease macrophage populations [20] significantly. 

The observed reduction of secreted mature IL-1β by curcumin is primarily attributed to the inhibition of NLRP3 inflammasome activation [21]. The mechanism of action can also be understood from two in vitro analytic studies that demonstrated a higher binding affinity of curcumin vs. nelfinavir to Mpro of SARS-CoV-2 [10,11,12,14]. This compound isolated from turmeric has been found to irreversibly inhibit aminopeptidase N/CD13, indicating that curcumin could play a role in preventing and decreasing coronavirus infection by inhibiting its cellular binding via CD13 [22]. 

As curcumin is not toxic even at high oral doses and is already approved and widely used in the food industry, its broad-spectrum anti-infective activity makes it a promising candidate. However, the relatively low availability and rapid metabolism of curcumin may limit its clinical impact. As it is insoluble in water at pH 7 and not stable in acid or alkaline solutions, the bloodstream’s net absorption is low, thus limiting systemic bioavailability. The potential benefits need to be tested in more robust in vitro and in vivo models, and advanced nanocarrier formulations may be needed to enhance its activity against viral pathogens [23]. 

### 3.2. Kaempferol

Background: Kaempferol is a flavonoid that can be obtained from several foods such as spinach, cabbage, kale, beans, tea, and broccoli and has been reported to have antioxidant and anti-inflammatory properties [24]. 

Evidence: Currently, it is under consideration as a possible cancer treatment and for antiviral bioactivities. Evidence for potential antiviral and antitumorigenic use derives from various Chinese herbal compounds with a high content of the flavanols kaempferol, kaempferol glycosides, and acylated kaempferol glucoside derivatives. Several studies have examined these flavanols’ potency to block the 3a ion channel formed by the ORF 3a-coded proteins, thus minimizing the viral production and release from the host cells. This ability gives the body a chance to adjust its immune system to counteract the viral attack. Specifically, kaempferol glycosides demonstrated a more potent inhibitory effect than kaempferol, highlighting the significance of glycosylation for antiviral activity [25,26]. 

Furthermore, docking analysis of kaempferol demonstrated its potential to inhibit viruses due to the high affinity, type, and amount of binding that occurs with the protein’s active site [10,27]. Pharmacological and docking analysis (China’s national guideline network) demonstrated that the kaempferol da-yuan decoction could bind with the ACE2 receptor and regulate the T-cell receptor [4]. 

A similar molecular blocking affinity to SARS-CoV-2 was also demonstrated in another Chinese study [25]. Therefore, the affinity of kaempferol binding to viruses appears higher compared with other flavonoid compounds. Kaempferol glycosides seem to be highly potent candidates for development as anticoronaviral agents. The ability to block the 3a ion channel and interfere with other steps of the viral life cycle emphasizes the importance of multitarget drugs [25] and could form the basis for developing new antiviral drugs with higher bioavailability. In vivo mice models suggest a protective effect, finding that kaempferol treatment attenuated pulmonary edema caused by influenza [24]. 

Considerations: The autoxidation process can limit kaempferol’s effects, so the doses need to be high and adjusted according to specific situations. Based on the antioxidant and free-radical-scavenging abilities of kaempferol and epidemiological studies that have demonstrated multiple biological effects [28], increasing the consumption of foods that contain kaempferol may prove to offer health benefits. 

### 3.3. Quercetin

Background: Quercetin is a flavonoid found mainly in onions, grapes, shallots, tea, and tomatoes, as well as many seeds, nuts, flowers, barks, and medicinal botanicals, such as *Ginkgo biloba*, *Hypericum perforatum*, and *Sambucus canadensis*. Its primary biological actions are antioxidant, anti-inflammatory, and antiviral, with some initial reports of anticancer effects. These actions are due to inhibition of lipid peroxidation, platelet aggregation, production of lipopolysaccharide-induced tumor necrosis factor in macrophages, and production of lipopolysaccharide-induced IL-8 in lung cells [28,29]. It is important to note that quercetin glycoside is much more efficient than other quercetin forms [30]. 

Evidence: The anti-inflammatory potential that can be expressed on different cell types, both in animal and human models, is based on modulatory, biphasic, and regulatory action on inflammation and immunity. Additionally, quercetin demonstrates an immunosuppressive effect on dendritic cell function, downregulating Th2-derived production of interleukin 4 (IL-4) [30,31]. Hydroquercetin and quercetin (found in onions and apples) have been demonstrated to function as zinc ionophores by chelating zinc and transporting it into the cell cytoplasm in an in vitro model. This function could theoretically enhance the antiviral actions of zinc [13]. The mechanism of action in vitro involves activated T cells with a decrease in IL-12-induced Th1 differentiation. In vivo animal experiments also support the anti-inflammatory effect. Quercetin ameliorates the inflammatory response induced by a high-fat diet and exerts a protective effect in mice by increasing cytokine secretion [32]. There is also evidence that quercetin can inhibit the growth and metastatic potential of melanoma cells in vivo in a mouse model. Quercetin at 25 and 50 mg/kg significantly delayed tumor growth and significantly decreased the number of metastatic lung colonies [2]. 

Some clinical results suggest that quercetin promotes anti-inflammatory and improved immunological outcomes. The results from a double-blinded, placebo-controlled, randomized trial showed that quercetin supplementation at 500 and 1000 mg/day for 12 weeks significantly increased plasma quercetin levels but had no influence on measures of innate immune function or inflammation in community-dwelling adult females [33]. On the other hand, another study associated quercetin with immunomodulatory effects reducing illness after intensive exercise. In this study, a dietary supplement of 1000 mg of quercetin three weeks before, during, and two weeks after a three-day period of 3 h of cycling in the winter resulted in a markedly lower incidence of URTI in well-trained subjects in the two weeks after the intensified training. This effect is applied to a broad spectrum of pathogens (including URTI-related rhinoviruses, adenoviruses, and coronaviruses). There was no effect on exercise-induced immune dysfunction, inflammation, and oxidative stress. In other studies of exercise-stressed athletes, a reduction in illness rates and a chronic augmentation of innate immune function was demonstrated [34,35].

Results have suggested anti-inflammatory and immune enhancement from in vitro (cells) and in vivo (animals). However, studies of quercetin supplementation in humans have demonstrated mixed results in boosting immune function. According to in silico data, quercetin showed a stronger affinity for SARS-CoV-2 protease and the ACE2 receptor than hydroxychloroquine [30]. Quercetin is likely safe to use both before and during COVID-19 infection, though it remains unknown whether it will provide a clinical benefit in decreasing virulence or illness symptoms. 

Considerations: Aspects to consider before choosing quercetin as a candidate include the following: (1) bioavailability, which is linked to metabolism and effects (for example, nanoparticles and polymeric micelles appear to contribute to a more sustainable release of quercetin), and (2) the careful evaluation of the target population and their health status [36]. 

### 3.4. Apigenin

Background: Apigenin is a flavonoid present in significant amounts principally in parsley, celery, onions, oranges, and herbs (*Matricaria chamomilla*). In vitro studies have shown that apigenin is active against DNA and RNA viruses, specifically HSV-1, poliovirus type 2, hepatitis C virus (HCV), hepatitis B virus, and adenoviruses (ADV). The antiviral activity appears to be related to nonglycosidic compounds, and hydroxylation at the 3-position is a prerequisite for antiviral activity. 

Evidence: Despite the numerous positive findings that have been reported on in vitro efficacy, less promising results have been obtained for most compounds in in vivo studies [2,36]. Antioxidant, antihyperglycemic, anti-inflammatory, and (in myocardial ischemia) antiapoptotic properties have been reported for apigenin. A recent review has summarized biological effects, such as cytostatic and cytotoxic activities toward various cancer cells, antiatherogenic and protective effects in hypertension, cardiac hypertrophy, and autoimmune myocarditis, representing additional potential health benefits. The apigenin mechanism of action is based on its modulatory effects on dendritic cells responsible for maintaining immune balance. 

In silico studies suggest that apigenin-7-glucoside may represent a potential inhibitor of SARS-CoV-2 Mpro [10]. The effect of apigenin on angiotensin-converting enzyme 2 (ACE2) has been tested; in hypertensive rats, ACE2 transcription and expression were upregulated in the kidney [37]. The ability to inactivate nuclear factor kappa-light-chain-enhancer or activate B cells sharply decreases interleukin 6, which in general acts as both a proinflammatory cytokine and an anti-inflammatory myokine, in lipopolysaccharide (LPS)-activated mouse macrophages [2,37].

Apigenin promotes different anti-inflammatory pathways, reducing COX-2 activity in human cell cultures. Its healing properties influence the metabolic pathway that affects the pharmacokinetic and tissue distribution. Liang et al. investigated the therapeutic effect of apigenin on neuroinflammation in the glial fibrillary protein interleukin 6 in mice using both immunohistochemical and behavioral tests. Like quercetin, apigenin was investigated as an inhibitor of melanoma cell growth in a mouse model and was found to significantly delay tumor growth and reduce the number of metastatic lung colonies.

Considerations: Interest in the various biological activities of apigenin is growing, and it has led to the development of efficient methods of extraction from its natural sources. However, dietary amounts may be insufficient to support the healing effects shown in in vitro studies.

### 3.5. Monolaurin

Background: Emerging science around monolaurin identifies it as a potential candidate for antiviral and other widespread benefits. Dietary consumption of coconut oil has been associated with several health benefits, among them increased antibacterial, antifungal, antiviral, antiparasitic, antidermatophytic, antioxidant, and immunostimulant activities [38]. This broad spectrum of antimicrobial effects is attributed to its medium-chain fatty acids (MCFA), particularly lauric acid, the most represented in coconut oil. Lauric acid is converted to monolaurin in the human body, and in this form, it exerts the above-mentioned wide range of antimicrobial and antiviral effects. 

Evidence: Monolaurin has even been demonstrated to inhibit the production of staphylococcal exoproteins by interfering with membrane signal transduction at the transcription level and inhibiting growth, as well as eradicating the biofilm formed by clinical isolates of *Staphylococcus epidermidis*. Of note, topical use of coconut oil for skincare or massage in infants is a popular practice in Asian countries, and some studies have shown a potentially preventative effect of monolaurin against the skin and systemic infections when used topically in both adults and preterm infants [39,40,41,42]. 

### 3.6. Conclusion and Perspectives

This review highlights the potential of selected flavonoids for use as adjuvant therapeutic agents against viral infections, including SARS-CoV-2. Simultaneously, more detailed studies are necessary before the use of these compounds can be recommended as part of antiviral therapy.

In fact, several important limitations hamper the quality of the existing evidence and its translatability to humans. In vitro, cell-free, and cell-based studies are predominantly compared with studies on animal models. Often the extracts tested are not standardized or inadequately titrated. Furthermore, the high concentrations of pure compounds often tested may not reflect these compounds’ actual bioavailability in vivo. Moreover, adequate pharmacokinetic and toxicological studies may also be lacking.

Additional issues to be addressed will include the solubility of the compounds and the appropriate choice of the best delivery systems (e.g., liposomes, polymeric micelles, and nanosuspension), dosing, and bioavailability. Appropriate design of clinical trials to demonstrate a prophylactic or therapeutic effect in humans remains challenging. This would also be true for assessing the bioavailability and bioactivity of these compounds due to high interindividual variability and the various mechanisms by which their biological actions may impact human health [2]. 

In summary, the in vitro results for P/Ns are promising. However, further well-designed animal studies that investigate the mechanism of action, pharmacokinetics, and safety profile of plant complexes and their isolated bioactive compounds are still needed [43] before embarking on further human clinical trials. These trials could potentially confirm and translate preliminary experimental findings into recommendations for use in COVID-19 or other viral illnesses. 

## Figures and Tables

**Table 1:** Summary of the bioactive compounds selected and potential mechanisms of action.

Bioactive	Food	In Vitro Properties	Mechanism of Action
Curcumin	Turmeric *Curcuma longa*	Antioxidant Anti-inflammation Antifibrosis	Good binding energy affinity to Mpro. Inhibit aminopeptidase N/CD13. Reduction of the AT1 receptor, AT2 receptor upregulated. Decreased the populations of macrophages. Inhibition of NLRP3 inflammasome signaling, and consequently NFkB, TNF-a, IL-6, IL-1B, and IL-18 expression.
Kaempferol	Spinach Cabbage Dill Kale Beans Tea Broccoli	Antioxidant Anti-inflammatory Anticancer	Affinity, type, and amount of binding with ACE2. Block the 3a and inhibit the ion channel receptor and regulate T-cell receptor. 3C-like protease (3CLpro).
Quercetin	Onion Grape Onions Tea *Ginkgo biloba* *Hypericum perforatum* *Sambucus canadensis*	Antioxidant Anti-inflammatory Antiviral	Affinity against COVİD-19 protease and ACE2 receptor ionophore, chelating zinc and transporting it into the cell cytoplasm. Reduce NLRP3 inflammasome signaling and consequently NFkB, TNF-a, IL-6, IL-1B, and IL-18 expression. Downregulating Th-2-derived interleukin 4.
Apigenin	Parsley Celery Onion Oranges *Matricaria chamomilla*	Antiviral Antioxidant Anti-inflammatory Antihyperglycemic	Modulatory effects on dendritic cells responsible for maintaining immune balance. Angiotensin-converting enzyme 2 (ACE2). Decreasing levels of interleukin 6. Reducing COX-2.
Monolaurin	Coconut oil	Antimicrobial	Inhibition of growth of biofilm by *S. epidermidis*.

**Table 2:** Summary of the bioactive compounds selected and common foods and content.

Bioactive	Molecule	Common Food	Content
Curcumin	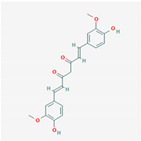	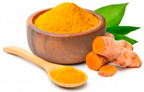	Turmeric: 3.14%
Kaempferol	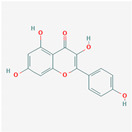	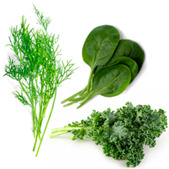	Spinach: 55 mg/100 g fresh weight Kale: 47 mg/100 g fresh weight Dill: 50 mg/100 g fresh weight
Quercetin	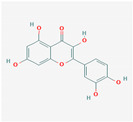	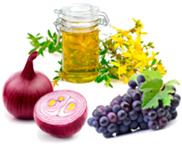	Onions: 45 mg/100 g fresh weight Dill: 79 mg/100 g fresh weight Grape (Merlot): 15 mg/100g
Apigenin	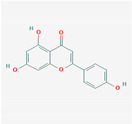	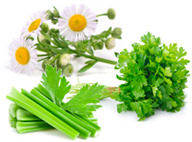	Parsley (dried): 45,035 μg/g Chamomile: 3000 to 5000 μg/g Celery seeds: 786 μg/g
Monolaurin	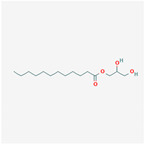	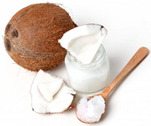	Coconut oil: 6%

Source: Structure and natural sources. Data from US Department of Agriculture and https://pubchem.ncbi.nlm.nih.gov/compound/.
